# Structure and dynamics of the Pacific North Equatorial Subsurface Current

**DOI:** 10.1038/s41598-020-68605-y

**Published:** 2020-07-16

**Authors:** Xiang Li, Ya Yang, Rui Li, Linlin Zhang, Dongliang Yuan

**Affiliations:** 1 Key Laboratory of Ocean Circulation and Waves, Institute of Oceanology, Chinese Academy of Sciences, and Function Laboratory for Ocean Dynamics and Climate, Pilot National Laboratory for Marine Science and Technology (Qingdao), 7 Nanhai Road, Qingdao, 266071 China; 2 Center for Ocean Mega-Science, Chinese Academy of Sciences, Qingdao, China; 3 University of Chinese Academy of Sciences, Beijing, China

**Keywords:** Physical oceanography, Physical oceanography

## Abstract

The North Equatorial Subsurface Current (NESC) has recently been found to flow westward below the North Equatorial Countercurrent in the subsurface layer across the Pacific Ocean. The structure, water mass properties, and the dynamics of the NESC are studied using Argo profiles and geostrophic currents, combined with moored current meter observations. The mean westward geostrophic currents of the NESC has been validated with moored current meter measurements at 4.7° N, 142° E in the far western tropical Pacific Ocean. Sizable seasonal-to-interannual variability of the NESC is indicated by the observations, with strong transports in boreal summer and during La Niña events, whereas weak transports in boreal winter and during El Niño events. The water masses of the NESC appear to be the mixture of the North and South Pacific intermediate waters, with the waters immediately below the thermocline closer to the North than to the South Pacific waters. A simulation using a linear continuously stratified model of ocean circulation suggests that the mean NESC is forced by wind curl through low baroclinic mode responses of the ocean.

## Introduction

The equatorial currents in the Pacific Ocean play an important role in the distribution of water mass and heat and freshwaters of the warm pool, which is key to the evolution of anomalous climate events, like El Niño/Southern Oscillation (ENSO). The zonal currents in the equatorial Pacific Ocean are of great scientific interests, the study of which has focused primarily on the surface currents in the past due to lack of observations in the subsurface ocean.

**Figure 1:**
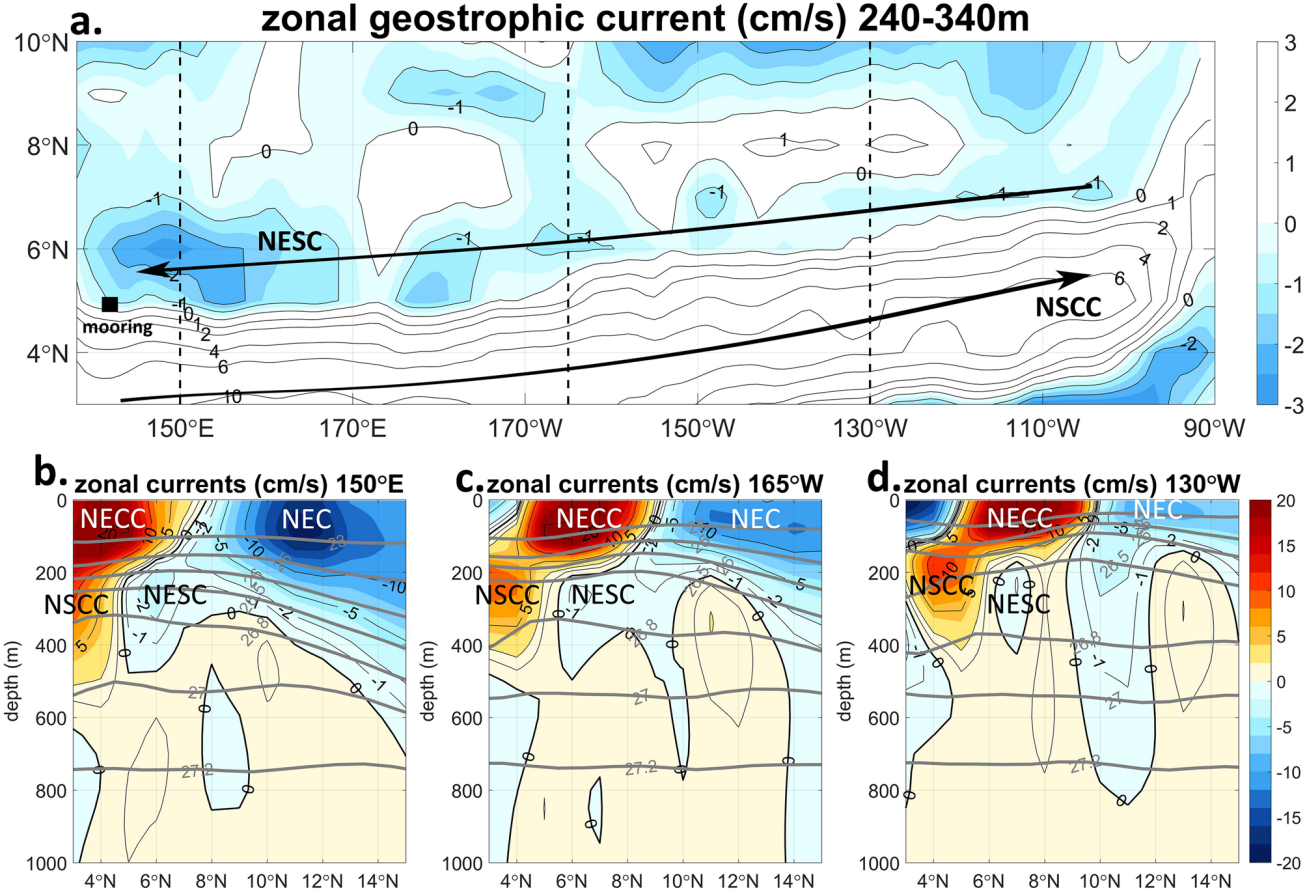
(**a**) Mean zonal Argo geostrophic currents vertically averaged between 240 and 340 m in the tropical North Pacific Ocean. The schematic axes of the major subsurface zonal currents, the Northern Subsurface Countercurrent (NSCC, a.k.a. Tsuchiya Jet) and the North Equatorial Subsurface Current (NESC) are marked. The mooring location and the meridonal sections of Argo profiles used in this paper (**b**–**d** and Fig. 4) are also marked by a solid square and dashed lines, respectively. Mean zonal Argo geostrophic currents in the meridional sections of (**b**) 150° E, (**c**) 165° E, and (**d**) 130° W in the western, central and eastern Pacific Ocean, respectively. The major zonal surface (North Equatorial Current, NEC; North Equatorial Countercurrent, NECC) and subsurface (NSCC; NESC) currents in the northern equatorial Pacific Ocean are marked in (**b**–**d**). The mean currents are averaged from the monthly Argo geostrophic currents from 2014 to 2018. The currents are averaged within longitudinal range of 5° close to the sections. The grey contours mark the potential density surfaces (minus 1,000 kg m^−3^). Unit of the density is kg m^−3^. The positive/negative values stand for eastward/westward currents. Only the westward currents are shaded in color to highlight the location of the NESC in (**a**). Unit of currents is cm s^−1^. The figure is generented by MATLAB software version R2019b (https://www.mathworks.com/).

Recently, a westward sub-thermocline current in the latitudinal range of 4° N–7° N (Fig. 1a) was found based on Argo geostrophic currents and ship-board acoustic Doppler current profiler (ADCP) measurements, which was named the North Equatorial Subsurface Current (NESC) by Yuan et al.^1^. The core of the geostrophic NESC is at the depths of 200–500 m, with velocity exceeding 2 cm s^−1^ and a mean westward transport reaching 4.2 Sv (1 Sv = 1 × 10^6^ m^3^ s^−1^). The existence of this current has been indicated by anecdotal evidence in some of the previous literatures: The Hawaii-to-Tahiti Shuttle Experiment^2^ showed a westward flow just north of the Northern Subsurface Countercurrent (NSCC, a.k.a. the Tsuchiya Jet) at 6° N–7° N under the North Equatorial Countercurrent (NECC) in the central Pacific Ocean between 150° W and 158° W at depths of 175–700 m, with a mean velocity of 4.2 cm s^−1^ and a mean transport of 2.5 Sv; The westward NESC also existed in the geostrophic currents at 7° N in the 125° W and 110° W sections in the eastern Pacific Ocean^3^, between 4° N and 8° N in the repeated hydrography sections along 137° E in the western Pacific Ocean^4^, and in compilation of shipboard ADCP measurements and Argo geostrophic currents across the equatorial Pacific Ocean^5^. The 8-month long mooring observations at 4.7° N, 140° E in 2014 have showed the existence of the NESC based on time series current measurements for the first time^6^.

The three-dimensional structure of the NESC has not been investigated systematically. In addition, the water mass properties, the variability, and the dynamics of the NESC, which are important for Pacific Ocean circulation and warm pool variability, have not been disclosed so far. In this paper, we use recent mooring measurements in the far western equatorial Pacific Ocean (Fig. 1a) and Argo profile measurements across the tropical Pacific basin to study the structure of this subsurface current. A linear continuously stratified model (LCSM) is used to investigate the dynamics of the NESC.

### Geostrophic currents

The Argo absolute geostrophic currents based on the P-vector calculation^1^ (equivalent to beta-spiral under the geostrophy and Bousinessq approximations) show the existence of the NESC across the entire basin of the tropical North Pacific Ocean (Fig. 1a). In the sub-thermocline layer between 200 and 500 m, the strong eastward NSCC lies south of 4° N in the western basin and moves slightly northward in the eastern basin. The NESC flows westward just north of the NSCC. The mean subsurface westward current is stronger and closer to the equator (5° N–7° N) in the west than in the east (6° N–8° N) (Fig. 1). The core geostrophic velocity of the NESC is larger than 2 cm s^−1^ in the western basin and 1 cm s^−1^ in the eastern basin. The subsurface westward NESC is surrounded by strong eastward currents of the NECC on the top and the NSCC in the south and can be easily distinguished from the westward North Equatorial Current (NEC) to the north at the surface (Fig. 1b–d). Thus, the connection of the undercurrent across the North Pacific basin beneath the NECC is unmistakable (see also meridional sections of Argo geostrophic currents in Ref.^1^).

**Figure 2:**
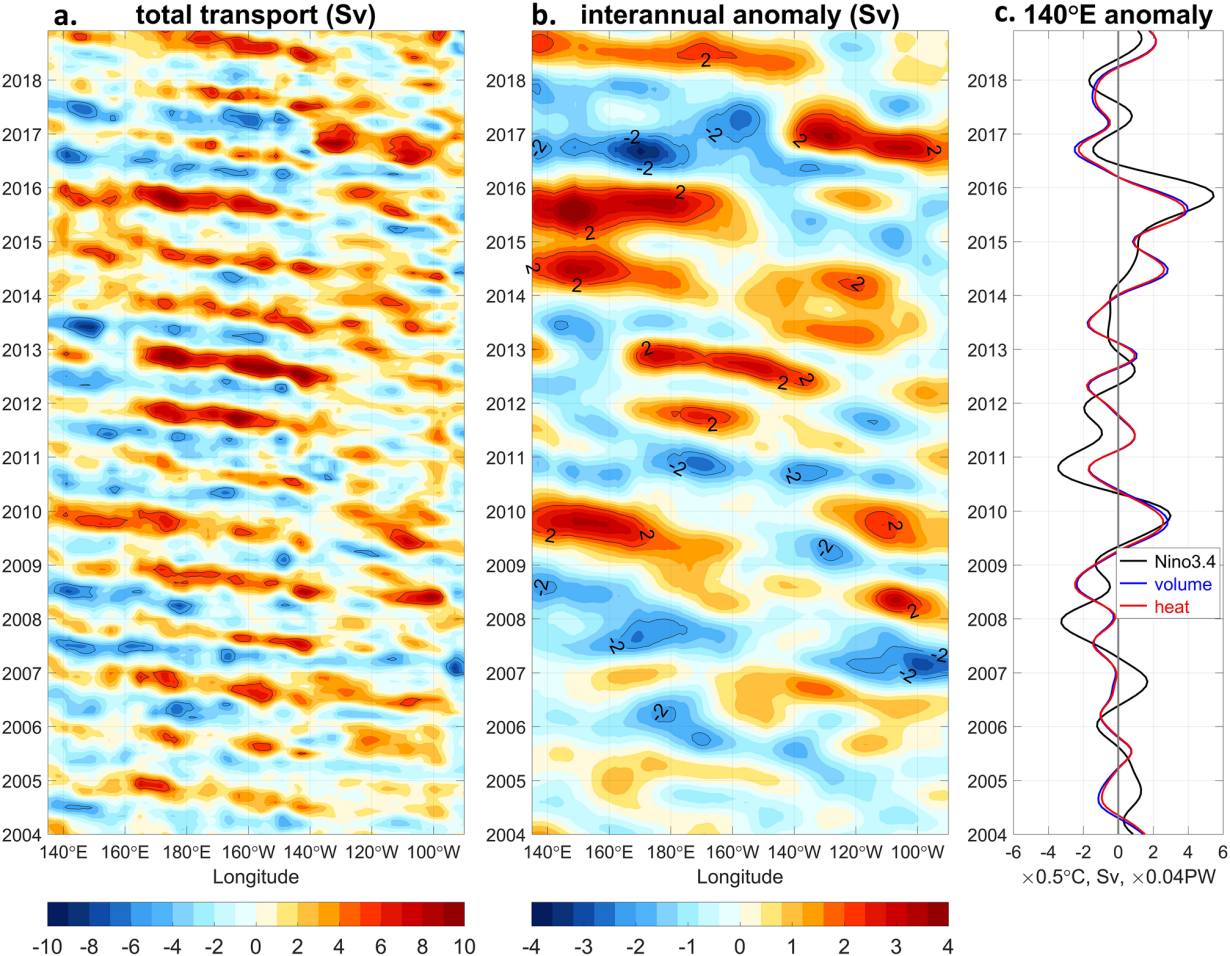
Hovmüller plot of the geostrophic volume transport within 1.5° latitudes from the axis of the NESC between 200 and 600 m (“Methods”) (**a**) and its interannual anomanlies in reference to the 2004–2018 climatology (**b**). (**c**) Comparison of the interannual geostrophic volume transport and the associated heat transport averaged between 135° E and 145° E, with the Niño 3.4 index. The interannual anomalies of the transports and the Niño 3.4 index have been low passed by a Butterworth filter with a cutoff period of 13 months. The total transport in (**a**) and its interannual anomalies in (**b**) are further smoothed by 3° and 10° longitudinal moving averages, respectively. Blue and red shadings indicate westward (negative) and eastward (positive) transports or transport anomalies, respectively. Black thin lines mark the 5 Sv and 2 Sv contours in (**a**) and (**b**), respectively. Unit of the volume and heat transports is Sv (1 × 10^6^ m^3^ s^−1^) and PW (1 × 10^15^ W), respectively. Unit of the Niño 3.4 index is degree Celsius. The figure is generented by MATLAB software version R2019b (https://www.mathworks.com/).

Variability of the NESC volume transport is investigated by integrating the zonal geostrophic currents between 200 and 600 m in the latitudinal range of ± 1.5° along the axis of the mean NESC (“Methods”) (Fig. 2a). The variability of the transport is weak in the eastern basin and becomes strong in the central and western basins, with the maximum westward transport exceeding 5 Sv frequently in the far western equatorial Pacific Ocean, where two low-latitude western boundary currents, the Mindanao Current and the New Guinea Coastal Current/Undercurrent (NGCC/UC) from the northern and southern hemispheres, respectively, meet slightly north of the equator^7^. West of 145° E, the NESC is the only sub-thermocline equatorial current flowing westward toward the entrance of the Indonesian seas^1^, countering the strong eastward NECC and NSCC current systems.

The NESC shows sizable seasonal-to-interannual variability and dominant westward propagation of the variability (Fig. 2a, b). The NESC in the western Pacific Ocean flows strongly to the west in boreal summer through fall and weakens or reverses in boreal winter. The westward propagation of the annual cycle of the transport is consistent with the westward and downward propagation of baroclinic Rossby waves^8^, the dynamics of which are studied in a separate paper^9^. The interannual variations of the NESC transport reach as large as 4 Sv in the far western Pacific Ocean (Fig. 2c), with interannual heat transport anomalies as large as 0.15 PW (1 PW = 1 × 10^15^ W), comparable to the total net surface heat flux of the entire warm pool^10^.

The low-passed interannual anomalies of the NESC transport in the far western equatorial Pacific are correlated significantly (the correlation coefficient r = 0.65, above the 95% significance level based on Monte Carlo simulations) with Niño 3.4 index at near zero time lag (Fig. 2c). In general, the interannual anomalies of the NESC transport in the western Pacific Ocean are negative and positive during El Niño and La Niña, respectively (Fig. 2b, c). During the 2014 weak El Niño and the succeeding 2015–2016 strong El Niño, the NESC nearly disappeared in the western Pacific Ocean, with interannual anomalies of more than 3 Sv in summer and fall of 2014 and 2015. However, the interannual NESC transport is not always in phase with the Niño 3.4 index. For example, during 2007/2008 and 2011/2012 La Niña events, the NESC transport anomalies were small, suggesting potential non-ENSO forcing of the interannual NESC, the dynamics of which need further investigation in a separate study.

### Mooring observations

**Figure 3:**
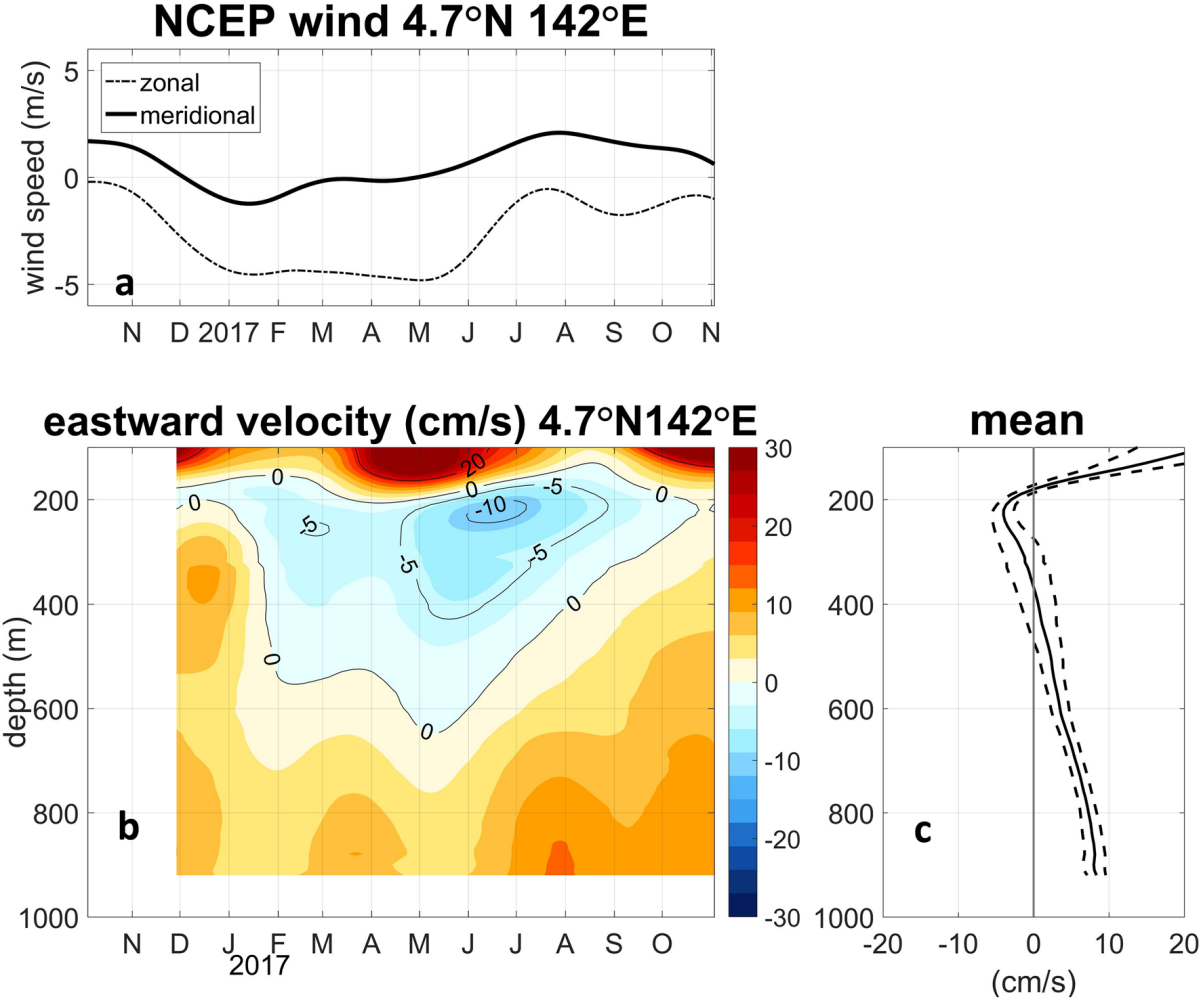
(**a**) Wind speed components of the NCEP/NCAR reanalysis, (**b**) zonal velocity of the moored current meter measurements, and (**c**) its mean zonal velocity profiles at 4.7° N, 142° E. Units of the wind speed and current are m s^−1^ and cm s^−1^, respectively. Positive values represent the northward component of the wind velocity and the eastward component of the current velocity. Dashed lines in (**c**) indicate the standard errors of the means at a 95% significance level. The figure is generented by MATLAB software version R2019b (https://www.mathworks.com/).

The moored current meter measurements show significant westward currents, sometimes larger than 10 cm s^−1^, in the depth range between 150 and 500 m at 4.7° N, 142° E (Fig. 3b). The westward current has been persistent during most of the mooring deployment and is only interrupted in boreal winter, which is consistent with the annual cycle of the Argo geostrophic subsurface zonal currents in the western Pacific (Fig. 2a), with westward and eastward maxima in boreal summer and winter, respectively. Sizable interannual variability may be included in the mooring measurements, as evidenced by the geostrophic anomaly currents (Fig. 2b). The subsurface current is not in phase with the local wind, suggesting remote forcing of the current (Fig. 3a, b). The westward propagation of the seasonal variability of the NESC volume transport (Fig. 2a) is consistent with the westward and downward propagation of the Rossby waves excited by the wind curl in the central-eastern Pacific^9^.

A mean westward flow between 180 and 350 m, below the eastward NECC, is indicated by the one-year mooring measurements at 4.7° N, 142° E (Fig. 3c). The mean velocity profile, with the mean NESC velocity larger than the standard error of the mean (Fig. 3c), is consistent with the geostrophic currents in the far western Pacific^1^ (see also Fig. 1b), showing a westward core of the mean NESC with the maximum velocity of 4 cm s^−1^ at the 230 m depth below the NECC with much stronger velocity (> 20 cm s^−1^) at the surface. The vertical extent of the mean NESC in the mooring measurements is a little smaller than that in the geostrophic currents, probably because the mooring location (4.7° N) is slightly away from the axis of the NESC (5° N–6° N) and close to the boundary between the NSCC and the NESC (Fig. 1a). The mean westward current does not extend to the intermediate layer below 800 m (Figs. 1b–d, 3c), consistent with the latest shipboard ADCP measurements^5^, showing that the NESC is distinct from the intermediate current systems down below at the 1,000 m Argo parking depth and at the deeper intermediate depths.

### Water masses

**Figure 4:**
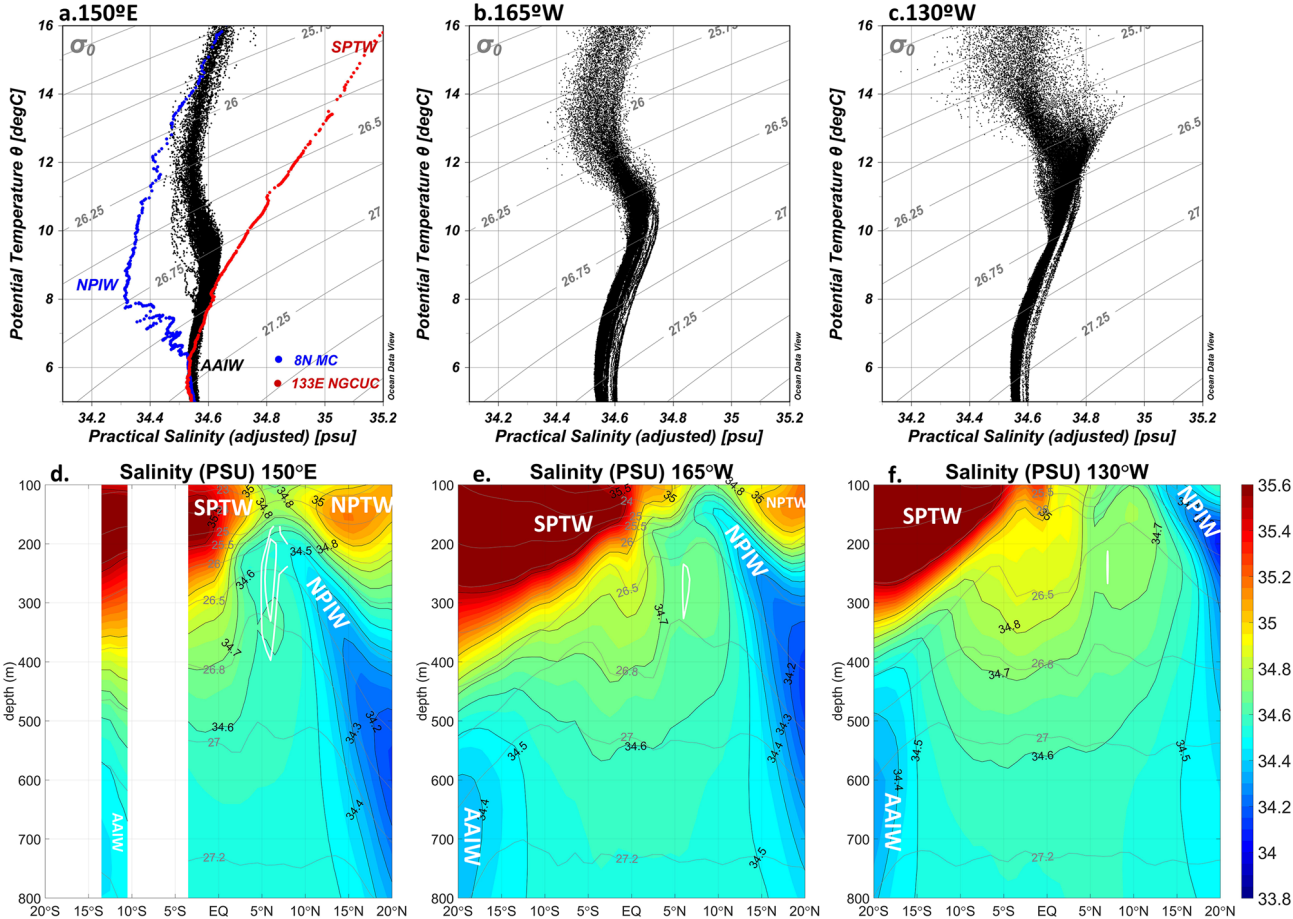
T–S relations of sea waters based on Argo profiles between 4° N and 6° N within 5° of longitude from 150° E in the western basin (**a**), and between 6° N and 8° N within 5° from 165° W and from 130° W in the central (**b**) and the eastern (**c**) basins. CTD station profiles at 8° N, 127° E and 0.45° S, 133.5° E representing the intermediate waters of the MC and NGCUC are overlapped in (**a**) to represent the typical water masses from the North and South Pacific. Salinity distributions (thin black contours with color shading) in meridional sections of 150° E (**d**), 165° W (**e**), and 130° W (**f**). In (**d**–**f**), the NESC cores are marked by the westward mean geostrophic velocity contours (white) at the interval of 1.0 cm s^−1^ and the density surfaces are plotted as thick grey contours. Units of the potential temperature, salinity, and potential density are degree Celsius, psu, and kg m^−3^, respectively. Salinity maxima of the North Pacific Tropical Water (NPTW) and South Pacific Tropical Water (SPTW), and salinity minima of the North Pacific Intermediate Water (NPIW) and the Antarctic Intermediate Water (AAIW) are marked in the panels (**d**–**f**). (**a**–**c**) is generented by Ocean Data View software version 5.1.0 (https://odv.awi.de/). (**d**–**f**) is generented by MATLAB software version R2019b (https://www.mathworks.com/).

The water masses of the NESC appear to be the mixture of the North and South Pacific waters according to the T–S relation of Argo profiles (Fig. 4a–c). In the equatorial Pacific Ocean, high salinity waters are transported across the equator by the NGCC/UC in the western Pacific and are carried to the east by the NSCC in the sub-thermocline, forming a salinity front between northern and southern Pacific water masses^11,12^. The water masses immediately north of the equator have a salinity maximum (> 35 psu) in the thermocline, which is called the South Pacific Tropical Water (SPTW) generated in the subtropical gyre of the South Pacific, and a salinity minimum (between 34.5 psu and 34.6 psu) at the intermediate depths, which is called the Antarctic Intermediate Water (AAIW) generated in the Southern Oceans^13^. In the North Pacific, the water mass with a salinity maximum (> 34.75 psu) is called the North Pacific Tropical Water (NPTW), which is generated in the subtropical gyre of the North Pacific^13^. In the intermediate depths in the North Pacific, the water mass with a salinity minimum (< 34.4 psu) is called the North Pacific Intermediate Water (NPIW), which is generated in the subpolar North Pacific^13,14^.

The NESC is located between density layers of σ_θ_ = 26.0 kg m^−3^ and σ_θ_ = 27.0 kg m^−3^ (Fig. 4d–f). The salinity distributions in the meridional sections across the equatorial Pacific suggest that the NESC is located at the southern edge of the NPIW, with the South Pacific waters of higher salinity immediately to its south. The fact that the salinity of the NESC is larger than those of the NPIW suggests that the NESC waters are a mixture of the northern and southern hemisphere waters. In the density layers above σ_θ_ = 26.5 kg m^−3^, the NESC salinity is closer to the NPIW salinity than to the SPTW salinity with a vertical salinity minimum especially in the western and central basin (Fig. 4a, b, d, e), suggesting the NPIW origin of the upper NESC waters. In the density layers between σ_θ_ = 26.5 kg m^−3^ and 27.0 kg m^−3^, the NESC salinity becomes a little higher than in the upper part, which lies between the salinity of NPIW and South Pacific lower thermocline waters, suggesting origins from both hemispheres. The westward decrease of the NESC salinity between σ_θ_ = 26.5 kg m^−3^ and 27.0 kg m^−3^ suggests more contribution of the NPIW during the westward movement of the NESC. Below σ_θ_ = 27.0 kg m^−3^, where the NESC nearly vanishes, the salinity is higher than intermediate water salinity in both hemispheres, probably due to mixing with surrounding waters during the movement of the water masses.

### Dynamics of the NESC

**Figure 5:**
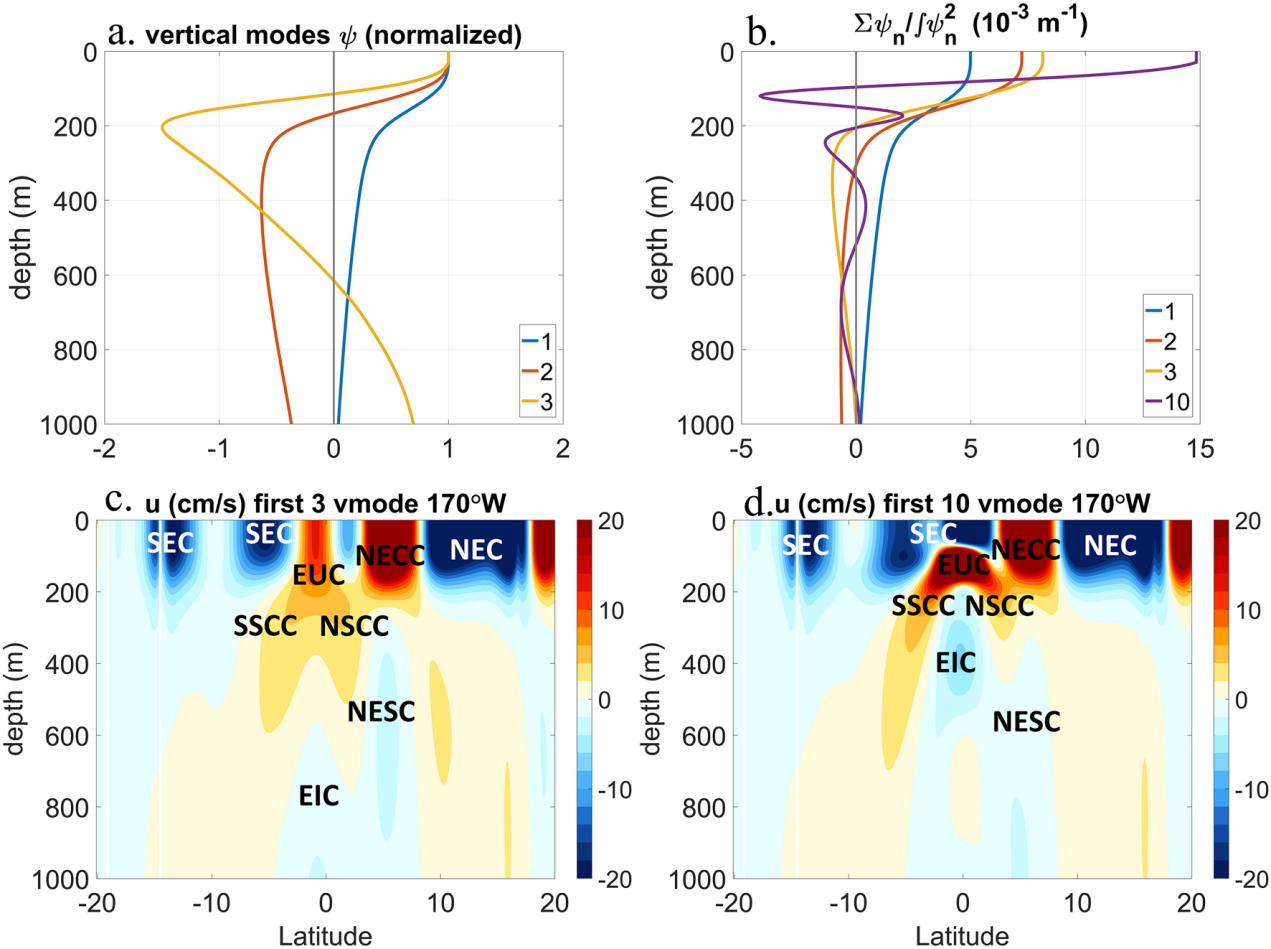
(**a**) Baroclinic modes functions (first three), and (**b**) progressive superpositions of the first 3 baroclinic modes functions scaled by equivalent depths compared with the superposition of the first 10 scaled baroclinic mode functions. The steady-state solution of the linear continuously stratified model (LCSM) in the meridional section of 170° W with first 3 (**c**) or 10 (**d**) baroclinic modes. The main zonal currents are marked in (**c**) and (**d**), including the North and South Equatorial Currents (NEC, SEC), the North Equatorial Countercurrent (NECC), the Equatorial Undercurrent (EUC), the Equatorial Intermediate Current (EIC), the Northern and Southern Subsurface Countercurrents (NSCC, SSCC), and the NESC. Colors stand for the magnitude of the zonal velocity, blue for the westward currents and red for the eastward currents, respectively. The baroclinic modes functions are normalized by values at the surface. Unit of the zonal velocity is cm s^−1^. The figure is generented by MATLAB software version R2019b (https://www.mathworks.com/).

The LCSM reproduces the main zonal currents of the mean surface circulation of the tropical Pacific Ocean successfully, using as few as 3 or 10 baroclinic modes (Fig. 5). The subtropical and tropical gyres enclosed by the main zonal currents, like the NEC between 8° N and 18° N, the NECC between 4° N and 8° N, and the South Equatorial Current (SEC) south of 4° N, are all simulated successfully. The zonal currents of the 3-mode and 10-mode simulations are slightly different near the equator in the central equatorial Pacific. The SEC in the surface layer of the 10-mode simulation is more realistic on the equator, since observations suggest that the SEC is south of and on the equator^2,5^.

Several subsurface currents are identified, such as the Equatorial Undercurrent, the North Equatorial Undercurrent, the NSCC, etc. flowing eastward, and the Equatorial Intermediate Current and the NESC flowing westward. The simulated NESC locates just below the NECC, with the core velocity exceeding 2 cm s^−1^ westward. The simulations using the first 3 and 10 vertical modes are similar in patterns, except for the currents in the vicinity of the equator. The successful simulations of the undercurrents by the LCSM, including the NESC, suggest that the undercurrents are generated by low baroclinic mode responses of the ocean circulation to the wind and wind curl forcing (Fig. 5c, d).

If ignoring Rayleigh friction and Newtonian cooling, all of the steady solutions of the baroclinic mode Shallow Water Equations (SWEs) are the same, except for the wind stress forcing multiplied by the coefficient $\frac{1}{\int {\psi }_{n}^{2}dz}$, where ψ_n_ is the n-th order baroclinic mode function. The vertical structure of the total steady solution of the linear continuously stratified ocean model is the superposition of the baroclinic mode functions multiplied by the scaling coefficient. The superposition of the first three scaled baroclinic mode functions shows the existence of undercurrents below the surface currents (Fig. 5b), due to the zero crossings near 200 m in the second and the third baroclinic modes (Fig. 5a). The stronger the surface current, the stronger the undercurrent is generated due to the baroclinic responses to the wind curl forcing. In reality, the baroclinic modes of very high orders are weakly forced by winds and strongly dissipated by friction, except on the equator, where they are damped by the Newtonian cooling to generate the Yoshida jets^15^. Therefore, the dynamics of the off-equatorial undercurrents are the responses of the low order baroclinic modes to the wind curl forcing, which drives the NEC and NECC at the surface.

The undercurrents below the NEC named the North Equatorial Undercurrent have been suggested to be generated by the converging potential vorticity fluxes of meso-scale eddies which is associated with the first mode baroclinic Rossby wave triad interactions in the eastern Pacific^16,17^. The theory predicts alternating zonal jets in the surface, with stronger jets in the eastern Pacific Ocean than in the west. In comparison, the simulated NESC and other sub-thermocline currents by the LCSM in this study are in the subsurface and increase from the east to the central-western basin (figure omitted), which are all in agreement with the observations.

In this paper, we focus on the dynamics of the mean NESC. The seasonal variability of the NESC in the western and central Pacific is also reproduced well by the LCSM, the dynamics of which are due to westward and downward propagation of baroclinic Rossby waves from the central and eastern Pacific^9^, the study of which will be reported in another paper.

## Conclusion

In this paper, the structure, water mass properties, and dynamics of the NESC are investigated. Argo geostrophic currents and direct measurements from a subsurface mooring in the western Pacific show the basin-wide connection of the NESC and its zonal extension to the far western Pacific Ocean. The NESC are found in the depth range between 200 and 500 m and in the density range between σ_θ_ = 26.0 kg m^−3^ and 27.0 kg m^−3^ along 5° N–7° N across the equatorial Pacific. The volume transport and the variability of the NESC increase from the east to the west, the phasing of which is consistent with westward propagation of baroclinic Rossby waves. The NESC is strong in boreal summer and La Niña and weak or absent in winter and El Niño in the far western Pacific Ocean. Interannual variability of the NESC transport reaching a magnitude of 4 Sv in the western Pacific was found during the 2015/2016 strong El Niño, which nearly obliterated the summer westward maximum of the NESC.

The water mass of the NESC is mixture of the northern and southern hemisphere waters in the sub-thermocline across the salinity front in the equatorial North Pacific Ocean. The upper part of the NESC comes mainly from the NPIW, whereas the lower NESC is from both hemispheres.

Study using a linear continuously stratified model (LCSM) shows that the dynamics of the mean NESC are low baroclinic mode responses of the ocean to wind curl forcing.

## Methods

### Mooring data

Ocean current measurements from a deep mooring are used in this study. The moorings were deployed at 4.7° N 142° E in the western equatorial Pacific Ocean in November of 2016, which roughly locates near the axis of the NESC (Fig. 1a). The observation lasted for about one year through October 2017. Two 75 kHz ADCPs were mounted on the main float at a nominal depth of 450 m looking upward and downward to measure the currents in the depth range of 50–950 m.

The bin size and the sampling interval of ADCP are 8 m and 1 h respectively. The quality-controlled hourly data are filtered by a Butterworth low-pass filter with a cut-off period of 120 days for further analysis.

### Argo data

The gridded Argo monthly data from 2004 to 2018 are downloaded from the International Argo center website https://www.argo.ucsd.edu/Gridded_fields.html. The gridded data include temperature and salinity profiles on a 1° longitude × 1° latitude horizontal grid with 58 vertical levels from 2.5 to 1975 dbar. Monthly absolute geostrophic currents are calculated using the P-vector inverse method^1,18^. The P-vector method is under the assumptions of geostrophy, Boussinesq approximation, and conservation of potential density and potential vorticity. No motionless level is assumed in the geostrophic current calculation. The volume transport variability of the NESC is estimated by integrating the zonal geostrophic currents between 200 and 600 m in the latitudinal range of ± 1.5° from the axis of the mean NESC. The axis of the NESC is defined as the latitudes where the vertically integrated velocity between 5° N and 7° N is the maximum westward. The interannual anomalies of the geostrophic transport are calculated based on the 2004–2018 climatology and low passed by a 4th-order Butterworth filter with a cutoff period of 13 months. The individual Argo temperature and salinity profiles in the NESC region are selected and downloaded at the Argo Data Management website https://www.argodatamgt.org/ for the water mass analyses.

### Ocean model

A linear continuously stratified model (LCSM) of ocean circulation is used to simulate the equatorial circulation in the Pacific Ocean. The model is the same as that used by Yuan and Han^19^ and Yuan et al.^20^, which follows the theory of McCreary^21^. The climatological mean density profile of the World Ocean Atlas 2013 version 2 (WOA13v2) averaged between 10° S and 10° N of the Pacific Ocean is used for vertical mode decomposition. The eigenfunctions (baroclinic mode functions) are not sensitive to the density profiles averaged over the different latitudinal bands of the equatorial Pacific Ocean. The model domain covers the tropical Indo-Pacific Ocean between 30° S and 30° N. The shallow water equations (SWEs) of each baroclinic mode are solved on a 0.1° longitude by 0.1° latitude horizontal grid with a real land-sea mask at the 200 m isobaths interpolated from the 1-min Gridded Global Relief Data (ETOPO1). The model is forced by the mean wind stress averaged from the monthly ERA interim data from 1979 to 2017. The integration starts from a motionless state and is spun-up for 100 years.

The SWEs of each baroclinic mode are forced by the same mean wind stress divided by the equivalent depth ${H}_{n}=\int {\psi }_{n}^{2}dz$, where ψ_n_ is the baroclinic mode function for mode *n*. The horizontal Rayleigh friction and Newtonian cooling coefficients are set at 1.3 × 10^–8^ m^2^ s^−3^, which are scaled by the inverse of the baroclinic wave speed squared in the SWEs. The horizontal viscosity coefficient is 400 m^2^ s^−1^, the same as that used in Yuan et al.^19^, which is small enough not to affect the solutions significantly.

### Other data

NCEP/NCAR reanalysis 1 wind vectors and Niño 3.4 index are downloaded from NOAA ESRL Physical Science Laboratory (https://www.esrl.noaa.gov/psd/data/reanalysis/reanalysis.shtml; https://psl.noaa.gov/gcos_wgsp/Timeseries/Nino34/). Two CTD station profiles obtained by R/V Kexue-1 and Baruna Jaya VIII are used to represent the typical T–S curves of the North and South Pacific water masses in the low-latitude boundary currents.

## Data Availability

The mooring data used in this study can be accessed at the web page https://itf.qdio.ac.cn/xzlxz/.
